# Behavioral Determinants of Leadership Engagement in Health Profession Students: A Cross-Sectional Study Using the Theoretical Domains Framework (TDF)

**DOI:** 10.2147/JHL.S525278

**Published:** 2025-10-04

**Authors:** Alla El-Awaisi, Menatallah Rayan, Dhabya Mohamed Al-Khater, Derek Stewart, Mohammed Al-Hamdani, Myriam Jaam, Mohammad Issam Diab

**Affiliations:** 1 Clinical Pharmacy and Practice Department, College of Pharmacy, QU Health, Qatar University, Doha, Qatar; 2 Hamad Medical Cooperation, Doha, Qatar; 3 Department of Public Health, College of Health Sciences, QU Health, Qatar University, Doha, Qatar

**Keywords:** health professions education, student leadership, behavioral determinants, interprofessional collaboration, leadership development, interprofessional education

## Abstract

**Introduction:**

Leadership is a critical competency in healthcare, influencing patient outcomes, organizational performance, and interprofessional collaboration. The study aimed to explore the behavioral determinants of leadership engagement among health profession students, and to examine associations with sociodemographic characteristics and leadership experience.

**Methods:**

A cross-sectional survey was administrated to health profession students at Qatar University health sector (n= 225). The survey was developed using the Theoretical Domains Framework (TDF) and analyzed using principal component analysis (PCA) to identify key behavioral components. Ordinal regression was used to assess associations between the components and sociodemographic factors, including nationality, college affiliation, and prior and current leadership experience.

**Results:**

PCA identified four behavioral determinants of leadership: 1) intention and self-efficacy, 2) awareness of opportunities, 3) perceived benefits, and outcomes, and 4) perceived barriers, each demonstrating acceptable internal consistency. Regression analysis confirmed significant predictive power across these determinants, with factors such as college affiliation, nationality, and prior leadership experience influencing outcomes. Approximately 25% of students reported holding leadership roles, yet more than half of these student leaders had no formal leadership training.

**Discussion:**

Findings highlight the importance of integrating structured leadership training and increasing access to leadership opportunities in a diverse, interprofessional student population. Initiatives such as interprofessional education student associations provide a valuable platform for fostering leadership skills in collaborative settings. These findings can inform the design of targeted educational interventions that support the development of essential leadership skills and cultivate the next generation of health leaders.

## Introduction

Leadership in healthcare has emerged as a critical competency, with evidence linking effective leadership with improved health outcomes, such as patient safety, patient satisfaction, interprofessional collaboration, organizational performance, and staff well-being.^1–3^ Recent advances in healthcare service delivery reflect a growing shift from individual-focused leadership models to collaborative approaches that prioritize shared leadership within interprofessional teams.^1^,^3–6^ In these settings, each team member is expected to assume a leadership role when professional expertise is required.^7^ Collaborative leadership is highlighted as one of the six competency domains by the Canadian Interprofessional Health Collaborative Framework emphasizing its significance in advancing effective interprofessional practice.^8^

Despite the importance of leadership development in healthcare, many existing models frequently focus on business or organizational leadership and often lack relevance to healthcare contexts.^3^,^9^ Recognizing the benefits of early leadership training, there is a growing emphasis on incorporating leadership development in health profession education, as recommended by multiple international bodies^10–12^ including the Institute of Medicine,^10^ the American Society of Health-System Pharmacists,^11^ and the Association of American Medical Colleges,^12^ all advocating the integration of leadership training within health profession curricula. They emphasize the importance of developing leadership skills among health profession students from their early years, providing adequate leadership opportunities and experiences to prepare them for the complex and interprofessional nature of healthcare settings. In this context, student organizations are among the most important avenues for leadership development, offering practical experiences that cultivate essential competencies for future health leaders.^13^

Despite its growing importance, there is a scarcity of research on health profession student leadership, which ultimately makes it challenging to design and implement interventions to increase students’ involvement in leadership opportunities and provide them with the required training and support to succeed and develop as student leaders. A recent scoping review of curricular content and competency frameworks in undergraduate medical education in the United States (US) highlighted several challenges. These included a lack of comprehensive frameworks focused on medical leadership, no clear consensus on the delivery approach, and, in most cases, leadership courses offered only as electives.^14^ However, a significant limitation is that these leadership development programs are predominantly tailored to physicians with limited inclusion of other health professions. This lack of integration hinders the development of collaborative leadership skills and fails to emphasize the importance of interprofessional practices.^15^

Few studies have systematically examined the potential behavioral influences that facilitate students’ engagement in leadership or the barriers they encounter.^16–20^ However, the research on leadership development in health professions education has several limitations. First, studies have generally been conducted on a single subset of health profession students, mainly medical students. Second, many of these studies assume that physicians naturally assume leadership roles, reinforcing a physician-centric model of leadership that excludes other professions.^14^,^15^,^21^ Third, these studies have not employed theoretical frameworks of behavioral change to explore potential behavioral influences, limiting the robustness and relevance of their findings.

Moreover, leadership engagement challenges differ across health professions. Medical students often face pressure to assume leadership roles, which negatively impacts their confidence in their leadership abilities and knowledge.^21^ This lack of confidence, along with a perceived sense of superiority frequently associated with the medical profession, leads to insecurity and disengagement among other health profession students, particularly those in nursing, midwifery, and dentistry.^21^ These dynamics can create interprofessional tensions and discourage collaborative leadership development. Additionally, many leadership development initiatives are short-term, lacking longitudinal integration into curricula and often failing to reflect the team-based realities of healthcare. As a result, students may miss early, meaningful leadership experiences, particularly those outside medicine, leading to inequities in access to leadership training and role modelling. Addressing these challenges requires a nuanced understanding of the behavioral determinants that influence students’ engagement in leadership activities across the full spectrum of health professions.

The Theoretical Domains Framework (TDF) was selected as the conceptual model for this study due to its comprehensive and theory-informed approach to understanding behavior, and it was used to guide the development of the survey items. The TDF integrates 33 psychological theories and 128 explanatory constructs into a single framework comprising 14 domains, that was developed and validated by behavioral expert committee consensus.^22^ The domains are: knowledge, skills, social/professional role and identity, beliefs about capabilities, optimism, beliefs about consequences, reinforcement, intentions, goals, memory, attention, and decision processes, environment context and resources, social influences, emotion, and behavioral regulation.^22^ The framework offers a robust structure to assess cognitive, affective, and contextual influences on leadership engagement among health profession students. The TDF is increasingly used in health-related research to identify and assess factors that influence behavior and to inform the design of behavioral change interventions promoting evidence based practice.^23–25^

The objective of this study was to explore the behavioral determinants of leadership engagement among health profession students at Qatar University (QU), and to examine associations with sociodemographic characteristics and leadership experience. Using the TDF as a conceptual model, this study investigates the factors that may impact student leadership engagement and identifies key behavioral determinants. Additionally, the study explored the support needs that student leaders perceive as beneficial for enhancing their leadership development to promote effective leadership development in health profession education. The study aims to generate actionable insights into the behavioral influences on student leadership engagement and to support the development of targeted educational strategies that foster leadership across health professions.

## Methods

### Study Design and Setting

This study utilized a cross-sectional, web-based, self-administered questionnaire administered at Qatar University, the leading higher education institution in Qatar and the largest contributor to the country’s health profession graduates. The health sector at Qatar University (QU) encompasses Colleges of Pharmacy, Medicine, Dental Medicine, Nursing, and Health Sciences, which collaborate across health professions to enhance student development and promote interprofessional learning.^26^

### Study Population and Sampling

This study targeted all health profession students enrolled at QU (n=1,352) across the health professions colleges. To ensure comprehensive participation, a universal sampling approach was employed by distributing the survey to all QU Health profession students. Based on an alpha level of 5%, 95% confidence interval, and anticipated 50% response distribution, Raosoft^®^ was used to calculate a minimum sample size of 300. However, to account for the low response rates, the survey was distributed to all students in the health profession.

### Questionnaire Development and Administration

The draft questionnaire underwent iterative revisions based on feedback from the research team and external validators, including three faculty members with expertise in survey research and five alumni with prior student leadership experience, to assess content and face validity, ensuring the clarity, relevance, and coverage of key constructs. Amendments to the questionnaire were made regarding the organization, clarity, and technical issues identified with the survey platform. The questionnaire was subsequently pilot tested with five health profession students at QU, requiring no further adjustments. The final questionnaire consisted of 20 questions organized into six sections. Sections A and B addressed demographic characteristics and included 36 TDF-based items on behavioral determinants of leadership engagement, which were mandatory for all participants. These items were developed by the research team with reference to the Determinants of Implementation Behavior Questionnaire (DIBQ), a validated 100-item generic questionnaire based on the 14 theoretical domains of the TDF,^27^,^28^ and were contextualized for health profession students. Students with current or past leadership roles completed Sections C–E, which focused on leadership experience, attributes of successful leaders, and training needs. These sections were adapted, with permission, from the National Student Leadership Survey (NSLS), developed by Western Sydney University.^29^ The questionnaire was administered online using Survey Monkey©. An invitation email was distributed to to all health profession students at QU, accompanied by a participant information sheet that outlined the purpose of the study, the voluntary nature of participation, and assurances of data confidentiality. The self-administered questionnaire required approximately 15–20 minutes to be completed. Reminders were sent during the data collection period to encourage participation and maximize response rates.

### Data Analysis

The data were exported from SurveyMonkey^®^ to IBM SPSS^®^ (Statistical Package for the Social Sciences) version 27. The thirty-six TDF questionnaire statements were developed based literature review and the use of the TDF as a guide for item generation (content validity). The items were subjected to principal component analysis (PCA), an exploratory dimension reduction technique used to identify underlying behavioral constructs by reducing a large number of items to a smaller number of components while retaining the maximum variance in the data variation as possible, thereby facilitating data analysis.^30^ PCA was selected over confirmatory factor analysis (CFA) because the TDF statements were newly developed and not previously validated, and we did not have a pre-specified hypothesis about the number or structure of components. PCA was deemed more appropriate for exploring the factor structure of the dataset.

The data’s suitability for PCA was confirmed by examining the correlation matrix (provided in Supplementary File 1), with the majority of correlations exceeding 0.30 for items belonging to their respective component. Further, Bartlett’s Test of Sphericity was significant (p < 0.001), and the Kaiser-Meyer-Olkin (KMO) measure exceeded 0.6, indicating adequate sampling adequacy.^31^

A minimum of five cases per item was ensured. Components were extracted based on Kaiser’s criterion (eigenvalues > 1.0), scree plot inspection, and cumulative variance explained. Varimax rotation was used to enhance interpretability. Reliability (internal consistency) was assessed using Cronbach’s alpha, with values >0.70 was considered desirable^32^ and 0.6–0.8, acceptable.^33^

Component scores for the final four behavioral determinants were calculated by computing the average of the weighted values of the Likert-scale items (1=strongly disagree; 5=strongly agree), with negative items being reverse-scored. The Cronbach’s alpha was used to test the internal consistency of each component. The associations between past and current leadership roles with each component were assessed using ordinal regression while accounting for key sociodemographic variables after checking for the assumptions of ordinal regression, including multicollinearity tests and proportional odds. No VIFs were above two for any independent variable when tested against each ordinal scale, and the test of parallel lines yielded non-significant effects, indicating that the proportional odds assumptions hold. Therefore, ordinal regression was deemed feasible as an analytical approach. Parameter estimates with 95% confidence intervals were calculated.

### Ethical Approval

The study was approved by Qatar University Institutional Review Board (QU-IRB 1456-EA/21). Ethical considerations were outlined in the participant information sheet and in the introductory section of the questionnaire, emphasizing confidentiality and voluntary participation. Informed consent was implicitly obtained, as participants indicated their agreement by continuing with and submitting the completed questionnaire.

## Results

### Participants’ Characteristics

In total, 225 participants completed the questionnaire, with a response rate of 17%. A summary of participants’ characteristics is presented in Table 1. Most participants (73.4%, n = 165) were early undergraduate students aged 17–22 years (80.4%, n = 181). Nearly half of the participants (40.9%, n = 92) were from the College of Pharmacy, followed by health sciences (34.7%, n = 78), and medicine (20.4%, n=46), while dental medicine students participated the least (4.0%). Most participants were female (87.6%, n=197), Qatari (32.4%, n=32.4%), Egyptian (16.9%, n=38), and Pakistani (10.2%, n=23). Nearly half of the participants (40.9%, n=92) held a student leadership position before university, with class representative (n=39) and team/club leader (n=25) being the most common roles.

**Table 1:** Participants’ Characteristics (n = 225)

Characteristic	Frequency, n (Percentage, %)
**Age (years)**	
17-22	181 (80.4%)
23-28	37 (16.4%)
29-34	4 (1.8%)
35-40	3 (1.3%)
**Gender**	
Male	28 (12.4%)
Female	197 (87.6%)
**College**	
Medicine	46 (20.4%)
Pharmacy	92 (40.9%)
Dental Medicine	9 (4.0%)
Health Sciences	78 (34.7%)
**Level of Study**	
Early Undergraduate (Years 1–3)	165 (73.4%)
Late Undergraduate (Years 4–6)	42 (18.6%)
Graduate	18 (8.0%)
**Nationality**	
Qatar	73 (32.4%)
Egypt	38 (16.9%)
Pakistan	23 (10.2%)
Sudan	14 (6.2%)
Jordan	11 (4.9%)
Syria	12 (5.3%)
Palestine	10 (4.4%)
Yemen	8 (3.6%)
Other (United Kingdom, India, Iraq, Mauritania, Tunisia)	36 (16.0%)
**Student Leadership Role Prior to Enrollment in Qatar University**	
Yes	92 (40.9%)
No	125 (55.6%)
Missing	8 (3.6%)

Of the 225 responses, 59 (26.2%) answered “Yes” to having a current or previous student leadership role. The most common leadership roles were student representative at a committee (32.2%, *n*=19), president or vice president of a student association (28.8%, *n*=17), and class representative (28.8%, *n*=17), Most students (30.5%, *n*=18) occupied their leadership role for one semester. Analysis of the participants’ descriptions of the roles and responsibilities associated with their positions revealed that the key responsibilities mentioned most frequently were “communicating class feedback” (*n*=11), “planning and organizing events” *(n*=16), and “task allocation” (n = 8).

### Behavioral Determinants of Student Leadership

Examination of the initial component structure for the principal component analysis revealed seven components with a cumulative variance of 63.56% for the 36 items. However, 15 items were cross-loaded (loaded.34 or higher on two or more components). This warrants further analysis, where six-, five-, and eventually four-component solutions were implemented, resulting in reductions in the number of cross-loaded items to 10 cross-loaded items for the four-component solution that explained 52.29% of the variance. Next, sequential item deletion for cross-loaded items at a threshold of.32 on two more items. We started with the heaviest cross-loaders: those with the smallest difference in cross-loadings and those with a high cross-loading. We continued to delete cross-loaded items in this manner, eventually yielding 24 items (12 deleted items) with one cross-loaded item and an improvement in variance to 55.76%, with retained items ranging in loadings between.503 and.840, and an acceptable visual bend in the scree plot. The components, namely intention and self-efficacy, perceived benefits and outcomes, awareness of opportunities, and perceived barriers, yielded Cronbach’s alpha values of.88,0.86,0.81, and.61, respectively. The four-component solutions and their corresponding items are presented in Table 2, together with thr distribution of participants’ responses on a 5-point Likert scale.

**Table 2:** Ordinal Regression Model Predicting Intention and Self-Efficacy Based on Sociodemographic Factors and Leadership Experience

Variable	*B*	Std. Error	Wald	*df*	P*	95% Confidence Interval	
						Lower Bound	Upper Bound
**Age**							
17-22	1.619	1.607	1.015	1	0.314	−1.531	4.77
23-28	0.876	1.548	0.321	1	0.571	−2.157	3.91
29-34	0.569	2.100	0.073	1	0.786	−3.546	4.684
35-40	0			0			
**Gender**							
Male	−0.243	0.515	0.222	1	0.637	−1.252	0.767
Female	0			0			
**College**							
Medicine	0.334	0.496	0.453	1	0.501	−0.639	1.307
Pharmacy	0.804	0.404	3.954	1	0.047	0.012	1.596
Dental Medicine	0.545	0.836	0.425	1	0.515	−1.094	2.184
Health Sciences	0			0			
**Level of Study**							
Undergraduate	−0.749	0.805	0.865	1	0.352	−2.327	0.829
Graduate	0			0			
**Nationality**							
Qatar	−0.276	0.529	0.273	1	0.601	−1.313	0.76
Egypt	−0.755	0.579	1.703	1	0.192	−1.89	0.379
Pakistan	0.032	0.641	0.003	1	0.96	−1.224	1.289
Sudan	−0.688	0.724	0.902	1	0.342	−2.107	0.731
Jordan	0.094	0.898	0.011	1	0.916	−1.666	1.854
Syria	0.429	0.796	0.29	1	0.59	−1.132	1.989
Palestine	−0.974	0.832	1.372	1	0.242	−2.605	0.656
Yemen	0.985	0.909	1.173	1	0.279	−0.798	2.767
Other	0			0			
**Leadership role before QU**							
Yes	1.364	0.379	12.991	1	<0.001	0.622	2.106
No	0			0			
**Leadership role at QU**							
Yes	1.056	0.405	6.802	1	0.009	0.262	1.85
No	0			0			

Note: * Significance at p < 0.05.

**Table 3:** Ordinal Regression Model Predicting Perceived Benefits and Outcomes from Sociodemographic Characteristics and Leadership Experience

Variable	*B*	Std. Error	Wald	*df*	p*	95% Confidence Interval	
						Lower Bound	Upper Bound
**Age**							
17-22	−1.644	1.591	1.068	1	0.301	−4.761	1.474
23-28	−1.514	1.538	0.969	1	0.325	−4.528	1.501
29-34	1.092	2.063	0.28	1	0.597	−2.952	5.136
35-40	0			0			
**Gender**							
Male	−0.317	0.515	0.378	1	0.539	−1.326	0.693
Female	0			0			
**College**							
Medicine	0.596	0.492	1.467	1	0.226	−0.369	1.561
Pharmacy	0.509	0.397	1.647	1	0.199	−0.268	1.287
Dental Medicine	1.345	0.847	2.526	1	0.112	−0.314	3.005
Health Sciences	0			0			
**Level of Study**							
Undergraduate	0.551	0.791	0.484	1	0.487	−1	2.101
Graduate	0			0			
**Nationality**							
Qatar	−0.704	0.535	1.732	1	0.188	−1.752	0.344
Egypt	−1.242	0.581	4.563	1	0.033	−2.382	−0.102
Pakistan	−0.403	0.643	0.392	1	0.531	−1.663	0.858
Sudan	0.651	0.728	0.798	1	0.372	−0.777	2.078
Jordan	−2.731	0.93	8.618	1	0.003	−4.555	−0.908
Syria	0.823	0.8	1.058	1	0.304	−0.745	2.392
Palestine	−0.898	0.833	1.163	1	0.281	−2.531	0.734
Yemen	0.664	0.918	0.523	1	0.47	−1.136	2.464
Other	0			0			
**Leadership role before QU**							
Yes	0.043	0.347	0.016	1	0.901	−0.637	0.724
No	0			0			
**Leadership role at QU**							
Yes	0.523	0.392	1.781	1	0.182	−0.245	1.29
No	0			0			

Note: * Significance at p < 0.05.

The model fit for all four outcomes [Intention and Self-Efficacy: χ²(18) = 46.937, p<0.001; Perceived Benefits and Outcomes: χ²(18) = 34.749, p =0.010; Awareness of Opportunities: χ²(18) = 43.765, p <0.001; Perceived Barriers: χ²(18) = 45.870, p <0.001] was significant, which suggests that each model was effective in differentiating between the levels of each outcome based on the tested list of predictors. The Nagelkerke pseudo R-squares were moderate for all outcomes:0.273,0.210,0.242, and.263 for intention and self-efficacy, perceived benefits and outcomes, awareness of opportunities, and perceived barriers, respectively. As shown in Table 2, being part of the College of Pharmacy, having a leadership role before joining Qatar University, and holding a current leadership role at Qatar University were associated with higher scores on the intention and self-efficacy determinants of leadership. Table 3 shows that being a national in Egypt or Jordan was associated with lower scores on the perceived benefits and outcome determinants of leadership. Table 4 shows that being part of the College of Pharmacy and being a national of Yemen were associated with higher scores on awareness of opportunities determinants of leadership, while being male was associated with lower scores on awareness of opportunities determinants of leadership. Further, holding a current leadership role at Qatar University was associated with higher scores on the leadership determinant related to awareness of opportunities. Table 5 shows that having a leadership role before joining Qatar University was associated with higher perceived barriers and being a national of Yemen is associated with lower perceived barriers.

**Table 4:** Ordinal Regression Model Predicting Awareness of Leadership Opportunities From Sociodemographic Characteristics and Leadership Experience

Variable	*B*	Std. Error	Wald	*df*	P*	95% Confidence Interval	
						Lower Bound	Upper Bound
**Age**							
17-22	0.584	1.489	0.154	1	0.695	−2.334	3.501
23-28	0.306	1.441	0.045	1	0.832	−2.518	3.13
29-34	−0.236	1.929	0.015	1	0.903	−4.017	3.546
35-40	0			0			
**Gender**							
Male	−1.072	0.483	4.924	1	0.026	−2.019	−0.125
Female	0			0			
**College**							
Medicine	0.806	0.459	3.08	1	0.079	−0.094	1.706
Pharmacy	1.31	0.38	11.896	1	<0.001	0.566	2.055
Dental Medicine	−0.289	0.768	0.141	1	0.707	−1.794	1.217
Health Sciences	0			0			
**Level of Study**							
Undergraduate	−0.102	0.736	0.019	1	0.89	−1.545	1.342
Graduate	0			0			
**Nationality**							
Qatar	0.22	0.487	0.204	1	0.652	−0.734	1.174
Egypt	−0.42	0.531	0.626	1	0.429	−1.46	0.62
Pakistan	−0.611	0.593	1.064	1	0.302	−1.773	0.55
Sudan	0.136	0.664	0.042	1	0.837	−1.164	1.437
Jordan	−0.354	0.823	0.185	1	0.667	−1.967	1.259
Syria	0.009	0.729	0	1	0.991	−1.421	1.438
Palestine	−0.645	0.77	0.702	1	0.402	−2.155	0.865
Yemen	2.53	0.882	8.232	1	0.004	0.802	4.258
Other	0			0			
**Leadership role before QU**							
Yes	−0.562	0.327	2.957	1	0.085	−1.203	0.079
No	0			0			
**Leadership role at QU**							
Yes	1.376	0.375	13.464	1	<0.001	0.641	2.111
No	0			0			

*Note: Significance at p < 0.05.

**Table 5:** Ordinal Regression Model Predicting Perceived Barriers to Leadership From Sociodemographic Characteristics and Leadership Experience

Variable	*B*	Std. Error	Wald	*df*	P*	95% Confidence Interval	
						Lower Bound	Upper Bound
**Age**							
17-22	−0.197	1.569	0.016	1	0.9	−3.271	2.878
23-28	0.043	1.519	0.001	1	0.978	−2.935	3.021
29-34	−1.9	2.025	0.881	1	0.348	−5.868	2.068
35-40	0			0			
**Gender**							
Male	0.179	0.507	0.124	1	0.724	−0.814	1.172
Female	0			0			
**College**							
Medicine	−0.94	0.49	3.687	1	0.055	−1.9	0.02
Pharmacy	−0.358	0.392	0.833	1	0.362	−1.126	0.411
Dental Medicine	0.361	0.824	0.191	1	0.662	−1.255	1.976
Health Sciences	0			0			
**Level of Study**							
Undergraduate	−0.021	0.788	0.001	1	0.978	−1.565	1.522
Graduate	0			0			
**Nationality**							
Qatar	0.109	0.518	0.044	1	0.834	−0.907	1.125
Egypt	−0.233	0.564	0.171	1	0.679	−1.338	0.872
Pakistan	1.035	0.632	2.681	1	0.102	−0.204	2.274
Sudan	0.058	0.706	0.007	1	0.934	−1.326	1.442
Jordan	−0.212	0.872	0.059	1	0.808	−1.92	1.497
Syria	0.397	0.78	0.259	1	0.611	−1.132	1.927
Palestine	0.762	0.825	0.854	1	0.355	−0.854	2.379
Yemen	−2.056	0.903	5.178	1	0.023	−3.826	−0.285
Other	0			0			
**Leadership role before QU**							
Yes	1.56	0.361	18.721	1	<0.001	0.853	2.267
No	0			0			
**Leadership role at QU**							
Yes	0.679	0.388	3.067	1	0.08	−0.081	1.44
No	0			0			

Note: * Significance at p < 0.05.

Principal component analysis (PCA) yielded four-components solution based on the Kaiser criterion (eigenvalues >1) and the interpretability of the factor structure. These four components collectively explained 55.8% of the total variance in leadership engagement items. The first component accounted for 28.7% of the variance, the second for 11.1%, the third for 8.5%, and the fourth for 7.5%. Rotation using the Varimax method improved the interpretability of the component structure, with the rotated solution showing more evenly distributed variance across components (20.4%, 15.4%, 11.3%, and 8.8% respectively). These components were subsequently interpreted as reflecting intention and self-efficacy, awareness of opportunities, involvement and perceived benefits, and perceived barriers (see Table 6).

**Table 6:** Total Variance Explained by Principal Component Analysis (the Four Components)

Component	Initial Eigenvalues			Extraction Sums of Squared Loadings			Rotation Sums of Squared Loadings	
	Total	% of Variance	Cumulative %	Total	% of Variance	Cumulative %	Total	% of Variance
1	6.889	28.702	28.702	6.889	28.702	28.702	4.885	20.354
2	2.657	11.07	39.772	2.657	11.07	39.772	3.687	15.365
3	2.045	8.519	48.291	2.045	8.519	48.291	2.707	11.281
4	1.794	7.474	55.766	1.794	7.474	55.766	2.104	8.767

The rotated component matrix (Table 7) presents the item loadings for these four components. Items were retained and assigned based on their highest loading (≥0.4). Component 1, intention and self-efficacy, includes items related to confidence, motivation, and perceived social support for leadership. Component 2, perceived benefits and outcome, reflects the value students place on leadership experiences. Component 3, awareness, captures familiarity with leadership opportunities and how to access them. Component 4, perceived barriers, includes items related to time constraints and conflicts with academic responsibilities.

**Table 7:** Rotated Component Matrix of Student Leadership Engagement Items Based on Principal Component Analysis, with Mapping to TDF Domains

Item wording	Mapped TDF Domain	Behavioral Determinant Components			
		Intention and self-Efficacy	Awareness of Opportunities	Perceived Benefits	Perceived Barriers
I am fully aware of student leadership opportunities in my college.	Knowledge	0.093	0.154	**0.796**	0.014
I am fully aware of student leadership opportunities in my university.	Knowledge	0.110	0.115	**0.809**	−0.115
I am fully aware of training opportunities (eg workshops, seminars) related to student leadership.	Knowledge	0.123	0.051	**0.684**	−0.038
I am fully aware of how to apply for student leadership opportunities in my university.	Knowledge	0.094	0.147	**0.816**	−0.043
I have the necessary skills required of a student leader.	Skills	**0.700**	0.202	0.038	0.095
I am/would be unable to resolve conflicts among my teammates.	Beliefs about capabilities	−0.162	−0.066	−0.030	**0.503**
I am/would be able to evaluate my teammates’ performance and give constructive feedback.	Beliefs about capabilities	**0.503**	0.210	0.165	−0.086
Being involved in student leadership is/would be time-consuming.	Beliefs about Consequences	0.090	−0.168	−0.054	**0.578**
Being involved in student leadership distracts/would distract from academic responsibilities.	Beliefs about Consequences	0.103	−0.042	−0.114	**0.840**
Being involved in student leadership is/would help develop my interpersonal skills.	Beliefs about Consequences	0.202	**0.835**	0.019	−0.007
Being involved in student leadership is/would be of benefit to my college.	Beliefs about Consequences	0.197	**0.759**	0.106	−0.051
Being involved in student leadership is/would make students’ voices be heard.	Beliefs about Consequences	0.268	**0.714**	0.049	0.034
Being involved in student leadership is/would be of benefit to my future professional practice.	Beliefs about Consequences	0.202	**0.798**	0.164	0.033
I will get recognition from my college if I am involved in student leadership.	Reinforcement	0.173	**0.633**	0.209	−0.233
I will get recognition from my peers if I am involved in student leadership.	Reinforcement	0.216	**0.671**	0.117	−0.218
I intend to apply for/continue in student leadership positions.	Intentions	**0.733**	0.239	−0.038	0.027
Compared to my other student tasks, being involved in student leadership is/would be high priority.	Goals	**0.602**	0.162	0.130	0.012
I am/would be unable to prioritize between my academic and student leader responsibilities.	Memory, attention, and decision processes	−0.072	−0.041	−0.044	**−0.719**
I am/would be able to easily make decisions as a leader.	Memory, attention, and decision processes	**0.628**	0.127	0.079	0.034
Faculty members in my college think I should participate in student leadership.	Social influences	**0.683**	−0.028	0.186	−0.321
Other students think I should participate in student leadership.	Social influences	**0.765**	0.148	0.047	−0.026
Most people who are important to me (eg family, close friends) think I should participate in student leadership.	Social influences	**0.705**	0.148	−0.055	−0.029
I am/would be enthusiastic about being a student leader.	Emotions	**0.724**	0.275	0.070	0.202
I have/ can have a clear plan of how I will carry out my student leadership roles and responsibilities.	Behavioral regulation	**0.642**	0.113	0.264	0.077

**Note**: Bolded items are the primary loadings that fell under the respective component.

**Table 8:** Mapping of Relevant BCTs for Addressing Perceived Barriers in Student Leadership and Intervention Strategies

Domain	Perceived Barrier	Recommended Intervention Type	Example of Intervention
Beliefs about Capabilities	“I am/would be unable to resolve conflicts among my teammates.”	Verbal persuasion to boost self-efficacy	Provide conflict resolution and communication workshops, offering students tools and techniques to handle conflicts constructively and offer them verbal encouragement and feedback to improve self-confidence in conflict resolution skills.
Beliefs about Consequences	“Being involved in student leadership is/would be time-consuming.”	Pros and cons	Guide students through a “Pros and Cons” exercise, helping them evaluate both positive and negative aspects of time spent in student leadership. This can help balance their perspectives on the commitment required.
Beliefs about Consequences	“Being involved in student leadership distracts/would distract from academic responsibilities.”	Anticipated Regret	Facilitate discussions on “Anticipated Regret”, encouraging students to consider how they might feel later if they miss out on student leadership experiences versus if they overly prioritize it at the expense of academics. This can help them make decisions that align with their personal and academic goals.
Memory, Attention, and Decision Processes	“I am/would be unable to prioritize between my academic and student leader responsibilities.”	Prompts/cues	Implementing reminders or scheduling tools to assist students in managing academic and leadership tasks effectively.

### Perspectives on the Support and Training Required by Student Leaders

More than half of the student leaders (54.2%, *n*=32) declared that they had not received training in their roles. Those who received training commonly consisted of leadership training programs (n=5) and workshops (n=4). When rating the importance of different means of leadership development, formal leadership programs and self-guided reading on leadership were perceived as extremely important by the lowest proportion of students (22.0%, *n*=13 for both). Participation in student networks within the university was rated as extremely important by the largest proportion of students (39.0%, *n*= 23), followed by participation in student networks beyond the university and membership in professional leadership associations, both of which were rated as extremely important by 33.9% (*n*=20) of the students. From the content analysis, the key forms of suggested training mentioned by the participants most frequently included the need for student leadership training (n = 11) and increased awareness of leadership opportunities (n = 4).

## Discussion

### Statement of Key Findings

The aim of this study was to explore the behavioral determinants influencing health profession students’ engagement with leadership using TDF. This study identified key behavioral determinants influencing leadership engagement among health profession students at QU, specifically, intention and self-efficacy, perceived benefits and outcomes, awareness of opportunities, and perceived barriers. Each factor contributes to shaping students’ engagement in leadership roles and their likelihood of continuing to pursue leadership opportunities. Using the TDF as a conceptual model, this study highlights that active involvement in leadership roles is associated with greater self-efficacy and awareness of opportunities. Additionally, it identifies gaps in training and support, with students indicating the need for structured programs to enhance their leadership skills. These findings highlight the importance of targeted support for fostering leadership development in health professions education and validate the utility of the TDF as a conceptual model and support its application in educational research within diverse and interprofessional student populations.

### Interpretation of Findings

Self-efficacy in leadership or the belief in one’s capabilities to lead effectively,^34^ which is linked to one’s intention and how one chooses to act,^35^ are important determinants of leadership engagement. In this study, self-efficacy emerged as a significant correlate of leadership engagement with pharmacy students and with those in their current leadership roles, demonstrating higher self-efficacy. A possible reason for this heightened self-efficacy among pharmacy students is that the College of Pharmacy was one of the first health profession colleges established at Qatar University and has developed a robust student association, the Qatar Pharmacy Undergraduate Society (QPhUS), affiliated with the International Pharmaceutical Federation (FIP).^36^ This established leadership culture likely encourages pharmacy students to engage and develop confidence in their leadership roles. Furthermore, students with previous leadership roles also exhibited higher self-efficacy, albeit to a lesser extent than those currently in leadership positions, supporting previous research showing that prior leadership experience correlated highly with leadership self-efficacy.^37^ This suggests that while past experiences contribute to leadership confidence, active engagement in leadership roles is particularly important for sustaining high levels of self-efficacy and emphasizing the importance of practical experience in developing and maintaining leadership confidence. These findings align with Bandura’s (1997) theory that self-efficacy shapes an individual’s intentions, performance, and persistence in tasks.^34^ Students with high self-efficacy not only engage more actively in leadership but also contribute to collective efficacy within teams.^38^,^39^ These findings highlight the value of practical leadership experiences in building confidence and reinforce the need for structured leadership opportunities in the curriculum.

The perceived benefits and outcomes are also related to leadership engagement. Students viewed leadership roles as valuable opportunities for developing interpersonal skills, amplifying their voices, and positively impacting their college and future professional practices. Leadership involvement was also associated with recognition from both colleges and peers. These findings align with previous studies indicating that students are drawn to leadership roles because of perceived advantages such as skill development, professional networking, résumé building, and career preparation^18^,^19^ Together, which foster substantial personal and professional growth, cultivating essential skills such as creative problem-solving, appreciation for diversity, and a sense of belonging within the university community; these findings are consistent with this study’s outcomes.^40^ Awareness of opportunities was another key determinant, particularly among students in active leadership roles, indicating that engagement fostered a more comprehensive understanding of the available opportunities. Although participants perceived themselves as being aware of student leadership opportunities and responsibilities, many reported limited knowledge of how to apply for these roles. This aligns with prior research showing that awareness and involvement in student organizations are closely linked, reinforcing the importance of accessible leadership avenues, which have an impact on student leadership skills and development.^41^ This finding implies that establishing and promoting accessible avenues for initial leadership engagement could serve as a catalyst for fostering sustained involvement and increasing awareness. Furthermore, our study also found that previous leadership roles did not significantly impact current awareness of opportunities, indicating that ongoing involvement is essential for maintaining awareness of leadership opportunities. Perceived barriers have emerged as significant obstacles to involvement in leadership. These barriers align with previous research that identifies common obstacles for leadership involvement and developing leadership skills such as dense curricula, competing academic demands, family and work obligations, and perceived lack of relevance^17^,^19^,^42^ and TDF domains associated with these barriers (beliefs about capabilities, beliefs about consequences, and memory, attention, and decision processes) suggest that incorporating behavioral change techniques, such as verbal persuasion to boost self-efficacy, pros and cons analysis, anticipated regret, and prompts/cues, may effectively mitigate these challenges and foster practical leadership competencies among students, as shown in Table 8.^43^

Over half of the student leaders reported no formal training, with most participants remaining neutral or expressing dissatisfaction with their preparation for leadership roles, suggesting the need for structured support through formal leadership curricula. Participants’ low ratings of existing leadership programs may reflect the perception that current offerings are inadequate. Developing leadership programs based on their recommendations can enhance students’ satisfaction. Such training, coupled with practical experience, could better equip students to balance leadership roles with academic responsibilities, enhancing both leadership skills and the overall benefits of these experiences.^44^ This need is reinforced by findings from a mixed-methods study of medical student leaders, where 85% of participants felt that leadership skills should be formally taught, and identified student leadership roles as their primary source of experience.^16^ While formal leadership programs and self-guided reading were rated lower by participants, content analysis revealed a strong interest in structured training. This aligns with another study in which 66% of students expressed an interest in leadership development programs.^20^ Well-designed leadership development programs have been shown to have a positive impact on students’ leadership skills, increased preparedness for leadership,^45^ emotional intelligence,^46^ enhanced leadership skills,^47^ and ability to drive change.^48^

It is also important to acknowledge the potential influence of cultural context on students’ perceptions of leadership. Qatar, like many Arab countries, is characterized by a collectivist culture and hierarchical social structure, in which physicians are often placed at the top of the professional hierarchy.^49^ This context may shape how leadership is understood and enacted by students.^50^,^51^ Furthermore, health profession students at Qatar University come from a wide range of cultures and nationalities. This study revealed intriguing differences in leadership engagement across nationalities, suggesting that cultural and contextual factors may influence how leadership is perceived and practiced among students from diverse backgrounds. As Alsharif et al highlight, successful educational engagement in the Arab world requires sensitivity to cultural values and difference, which may shape attitudes towards leadership and professional identity.^50^

### Implications for Practice

Effective strategies to enhance self-efficacy and the intention to lead, particularly emphasizing the benefits of leadership, addressing barriers, and raising awareness of opportunities, can greatly enhance student leadership programs. Focusing on these factors, students are more likely to perceive leadership as attainable and worthwhile, ultimately increasing their engagement. Interprofessional education (IPE) initiatives present valuable opportunities for health profession students to strengthen their skills in interprofessional leadership, collaboration, and communication to prepare for collaborative practice.^21^,^52^ Integrating student leadership into both curricular and extracurricular IPE initiatives can create collaborative student leadership opportunities across health professions.^52^ Structured institutional support with clear guidance and mentorship is essential for student leaders to fully engage in and benefit from IPE experiences.^52^,^53^ The IPE Student Association at Qatar University provides a promising platform for advancing student leadership development within an interprofessional framework, fostering a collaborative environment in which students can develop and refine essential leadership skills across different health professions.^54^ Encouraging continuous involvement in campus and interprofessional activities will ultimately prepare students to lead effectively in future healthcare settings.

### Study Strengths and Weaknesses

A major strength of this study is its application of the TDF to explore behavioral determinants within a diverse cohort of health profession students, offering a comprehensive and theoretically grounded perspective on the factors that influence leadership engagement. This study also demonstrates how the TDF can be effectively operationalized to investigate leadership engagement in health professions education. While the TDF has been extensively used in implementation science and clinical behavior research, its application in educational settings, particularly in exploring professional behaviors such as leadership, is still emerging. By mapping questionnaire items to specific TDF domains and validating them through PCA, this study contributes a replicable model for behaviorally informed curriculum development and evaluation. Furthermore, the combination of PCA and reliability testing further strengthens the robustness of the findings by ensuring that the identified components are statistically sound and reliable. Additionally, this study addressed this critical gap by including students from multiple health professions, as previous research has often focused exclusively on medical students. However, a lower-than-anticipated response rate (17%) may limit the generalizability of the findings. To mitigate this, we used a universal sampling approach, sent follow-up reminders, and ensured anonymity to encourage honest responses and participation across student groups. Nonetheless, potential limitations remain, including for social desirability bias, recall bias, and varying levels of student interest in leadership. It is also possible that students with greater interest in leadership were more likely to respond, which may have led to an overestimation of leadership engagement and motivation. This self-selection bias should be considered when interpreting the results. Furthermore, this study’s focus on a single institution in Qatar which may limits the transferability of the findings to other settings and cultural contexts.

### Further Work

Future research should consider expanding the sample size and employing diverse data collection methods to enhance representativeness and allow for subgroup analysis. Designing a leadership development program informed by the areas of improvement identified in this study, by using a Delphi consensus process with expert leadership coaches, would be beneficial. This program can then be evaluated using a pretest–post-test experimental design to assess the impact on leadership skills and competencies. Furthermore, the study revealed intriguing nationality-based differences in leadership engagement, suggesting that cultural and contextual factors may influence the perceptions and practices of leadership among students from diverse backgrounds. These variations highlight the importance of considering cultural context when developing leadership training programs to meet the needs of a multicultural student population. Although the effect of nationality on behavioral determinants was evident, subgroup sample sizes for individual nationalities and for students reporting prior leadership experience were insufficient to support statistically robust comparisons. Future studies with larger subgroup samples are needed to examine these associations with adequate statistical power. Further qualitative exploration of these cultural and contextual differences could offer valuable insights into how students engage with leadership across varying cultural backgrounds. This approach would support the development of more culturally sensitive and contextually tailored leadership development programs. Further cross-cultural research is needed to explore how leadership is perceived and fostered in different cultural and institutional contexts.

## Conclusion

This study enhances understanding of the behavioral determinants that influence leadership engagement among health profession students by applying the TDF in a novel educational context. It offers a theory-informed perspective by identifying four core components: intention and self-efficacy, awareness of opportunities, perceived benefits, and perceived barriers that shape student leadership engagement. These findings add to the literature by contextualizing leadership engagement across multiple health professions, an area underrepresented in leadership education research. The findings emphasize the importance of structured training and accessible leadership pathways to build confidence and foster sustained engagement, thereby promoting inter-professional collaboration and equipping students with essential skills for future healthcare environments. Addressing these determinants through tailored leadership programs and integrating leadership opportunities into interprofessional education initiatives may empower students across health professions to develop key leadership competencies and prepare them to meaningfully contribute to patient-centered collaborative care. The study provides a useful foundation for designing longitudinal and interprofessional leadership training initiatives. Future research should examine how these behavioral determinants evolve during clinical training and into early professional practice. Embedding targeted, theory-informed interventions within interprofessional curricula may strengthen leadership readiness and better prepare students for collaborative, patient-centered healthcare environments.
